# Deaths Related to Hurricane Irma — Florida, Georgia, and North Carolina, September 4–October 10, 2017

**DOI:** 10.15585/mmwr.mm6730a5

**Published:** 2018-08-03

**Authors:** Anindita Issa, Kirtana Ramadugu, Prakash Mulay, Janet Hamilton, Vivi Siegel, Chris Harrison, Christine Mullarkey Campbell, Carina Blackmore, Tesfaye Bayleyegn, Tegan Boehmer

**Affiliations:** ^1^Epidemic Intelligence Service, CDC; ^2^Division of Environmental Health Science and Practice, National Center for Environmental Health, CDC; ^3^Florida Department of Health; ^4^Applied Epidemiology Fellowship Program, Council of State and Territorial Epidemiologists; ^5^Georgia Department of Public Health; ^6^North Carolina Department of Health and Human Services.

Three powerful and devastating hurricanes from the 2017 Atlantic hurricane season (Harvey [August 17–September 1], Irma [August 30–September 13], and Maria [September 16–October 2]) resulted in the deaths of hundreds of persons. Disaster-related mortality surveillance is critical to an emergency response because it provides government and public health officials with information about the scope of the disaster and topics for prevention messaging. CDC’s Emergency Operations Center collaborated with state health departments in Florida, Georgia, and North Carolina to collect and analyze Hurricane Irma–related mortality data to understand the main circumstances of death. The most common circumstance-of-death categories were exacerbation of existing medical conditions and power outage. Further analysis revealed two unique subcategories of heat-related and oxygen-dependent deaths in which power outage contributed to exacerbation of an existing medical condition. Understanding the need for subcategorization of disaster-related circumstances of death and the possibility of overlapping categories can help public health practitioners derive more effective public health interventions to prevent deaths in future disasters.

Hurricane Irma, a Category 5 hurricane (185-mph winds), caused catastrophic damage in the Caribbean before moving northwest and making landfall in Florida on September 10, 2017, as a Category 4 hurricane. Wind damage compromised power lines, and a storm surge caused extensive flooding, primarily along the coast. Irma affected the entire state of Florida; 7 million residents were evacuated (*1*) and 6.7 million utility customers lost power (*2*). As Irma traveled inland, it weakened to a tropical depression; despite this weakening, 75,000 customers in North Carolina (*3*) and >900,000 customers in Georgia (*4*) experienced storm-related power losses.

As part of CDC’s public health response to the hurricanes, the Epidemiology and Surveillance Task Force in the Emergency Operations Center tracked online media reports of hurricane-associated deaths and contacted states for confirmation (*5*,*6*). The Georgia Department of Public Health and North Carolina Department of Health and Human Services provided CDC with information on confirmed hurricane-related deaths. Concurrently, the Florida Department of Health identified deaths associated with Hurricane Irma through examination of vital statistics death data from the electronic death registration system, reports from the Florida Medical Examiners Commission, and media reports. To identify hurricane-related deaths, Florida used text-parsing algorithms to query “How Injury Occurred” and “Literal Cause of Death” fields on the death certificates. Researchers developed a circumstance-of-death categorization scheme based on previous research (*7*) and used it to classify Hurricane Irma–related deaths (Box). This report summarizes the circumstances of confirmed Hurricane Irma–related deaths from September 4 to October 10, 2017, in Florida, Georgia, and North Carolina and highlights the need for detailed analysis of disaster-related circumstances of death.

BOX **Categorization scheme used to classify circumstances of deaths associated with Hurricane Irma — Florida, Georgia, and North Carolina, September 4–October 10, 2017**

**Directly hurricane-related**

**Accident**

- Trauma from wind/rain-associated structural collapse, falling structures, or flying debris during storm
- Drowning or asphyxiation from rain/floods/landslides
  - Automobile-related
  - Boat-related
  - Residence/Building-related
  - Other or unknown mechanism
- Electrocution from lightning

**Indirectly hurricane-related**

**Natural**

- Hazardous environmental conditions (e.g., leptospirosis)
- Exacerbation of existing medical condition
  - Emergency medical issue inadequately addressed because of loss/disruption of emergency transportation services
  - Loss/Disruption of usual access to medical/mental health care (e.g., clinics, pharmacies)
  - Loss/Disruption of public utilities (e.g., electricity) needed for medical treatment (e.g., dialysis, oxygen, refrigerated medications, etc.)
  - Loss/Disruption of heat or cooling systems where excess heat/cold exacerbated preexisting medical conditions
  - Primarily induced by stress/anxiety before, during, or after the storm where access to medical services was available (e.g. myocardial infarction)

**Accident**

- Poisoning
  - Carbon monoxide
  - Industrial hazards
- Vehicular accident
  - Precipitated by hazardous road/traffic conditions
  - Not precipitated by hazardous conditions, but occurring while in route to or from hurricane-affected area (e.g., involving evacuation of disaster response/aid
- Preparation/Repair
  - Fall from roof, ladder, etc.
  - Sharp force injury during preparation/repair (e.g., chainsaw injury)
  - Electrocution while working on nonfunctional power line
- Loss/Disruption of emergency services (e.g., fire department)
  - Burn or smoke inhalation
- Hazardous or unfamiliar environmental conditions
  - Fall from standing height caused by inadequate lighting, storm debris in walkway, or unfamiliar environment
  - Collapse of unstable structures after storm
  - Electrocution from contact with downed power line

**Possibly hurricane-related**

**Homicide**

**Suicide**

**Undetermined**

**Source:** Categorization scheme based on report by Combs et al. (https://academic.oup.com/ije/article/28/6/1124/771525).

**Note:** Direct deaths are caused by environmental forces of the hurricane and direct consequences of these forces and indirect deaths are caused by unsafe or unhealthy conditions because of loss or disruption of usual services, personal loss, or lifestyle disruption; possibly related deaths include deaths attributed to the hurricane in which the indirect or direct relation of the death to the hurricane is not clear.

Among the 129 hurricane-related deaths identified in Florida, Georgia, and North Carolina, 123 (95.3%) occurred in Florida; 88 (68.2%) decedents were male, and the median age was 63 years (range = 1–99 years). Eleven (8.5%) deaths were directly related to the hurricane, 115 (89.1%) were indirectly related, and three (2.3%) were possibly related (Table).

**TABLE:** Circumstances of confirmed deaths* related to Hurricane Irma — Florida, Georgia, and North Carolina, September 4–October 10, 2017^†^

Circumstance of death	No. of deaths	% of total deaths^§^
**Directly hurricane-related^¶^**	**11**	**8.5**
Accident	11	8.5
Drowning related to flooding	7	5.4
Tree-related injuries	4	3.1
**Indirectly hurricane-related^¶^**	**115**	**89.1**
Natural	48	37.2
Existing medical condition exacerbation	46	35.7
Stress-related cardiac disease	23	17.8
Heat-related	17	13.2
Oxygen-dependent	3	2.3
Disruption of emergency medical services	3	2.3
Floodwater infection	2	1.6
Accident	67	51.9
Carbon monoxide poisoning	16	12.4
Preparation/Repair injury	15	11.6
Motor vehicle crash	13	10.1
Falls from standing height**	13	10.1
Other^††^	12	9.3
**Possibly hurricane-related^¶^**	**3**	**2.3**
Homicide	1	0.8
Suicide	1	0.8
Undetermined	1	0.8

* N = 129.

^†^ Among the 129 total deaths, 123 are from Florida. The mortality data are accurate as of July 16, 2018.

^§^ Might not sum to 100% because of rounding.

^¶^ Direct deaths are caused by environmental forces of the hurricane and direct consequences of these forces. Indirect deaths are caused by unsafe or unhealthy conditions because of loss or disruption of usual services, personal loss, or lifestyle disruption. Possibly related deaths include deaths attributed to the hurricane in which the indirect or direct relation of the death to the hurricane is not clear.

** Falls from standing height occurred in elderly persons. The word “hurricane” was recorded in the death certificates. Four of the 13 decedents died after the surveillance end date of October 10, 2017, from complications of falls that occurred during the hurricanes.

^††^ Includes deaths caused by drowning not related to flooding (n = 5) and collapse of unstable structures after the hurricane had passed (n = 1). Drowning not related to flooding includes persons found floating in swimming pools; these death certificates contain no mention of flooding.

The most common category of indirect circumstance of death was exacerbation of an existing medical condition (46; 35.7%) (Table[Fig F1]). Specifically, 23 (17.8%) deaths were associated with chronic health problems, such as cardiac disease, that were exacerbated by stress and anxiety related to the hurricane. Three (2.3%) deaths in chronically ill patients were attributed to disruption of emergency medical services. The remaining 20 (15.5%) deaths associated with exacerbation of an existing medical condition could also be categorized as power outage–related deaths (Figure). Seventeen (13.2%) heat-related deaths were associated with lack of air conditioning, and three (2.3%) deaths occurred in patients whose medical treatment (e.g., supplemental oxygen) was electricity-dependent. Fourteen (10.9%) of the heat-related deaths occurred among geriatric patients with existing chronic diseases who resided in an assisted-living facility in Florida that was without power for several days during a period of hot weather after the hurricane’s landfall. An additional 27 (20.9%) power outage–related deaths were not related to exacerbation of an existing medical condition. These included 16 (12.4%) carbon monoxide poisonings.

**FIGURE F1:**
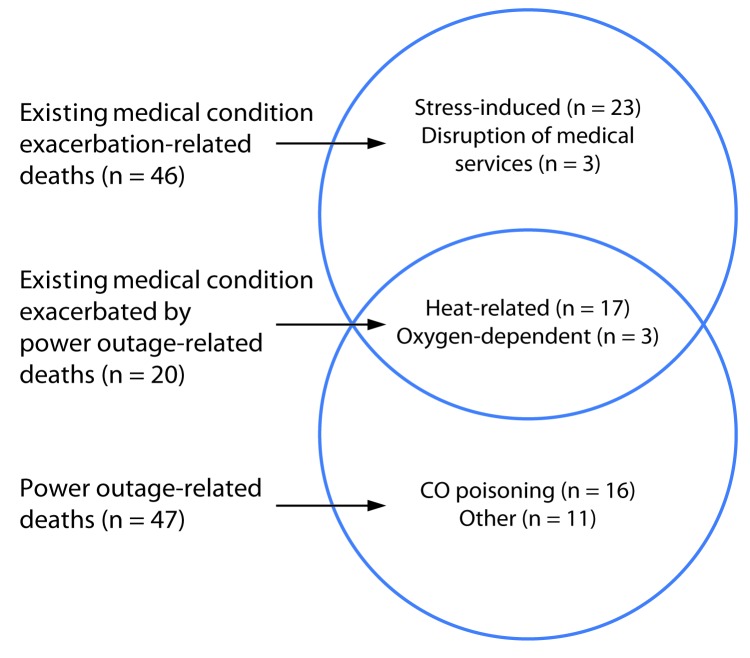
Overlapping circumstances of deaths associated with existing medical condition exacerbation and power outages caused by Hurricane Irma — Florida, Georgia, and North Carolina, September 4– October 10, 2017*^,†^ **Abbreviation:** CO = carbon monoxide. * Total number of deaths = 73. ^†^ Fourteen of the 17 heat-related deaths occurred in residents of an assisted living facility in Florida that was without power for several days.

## Discussion

Currently no published standardized methodology exists for analyzing disaster-related mortality data to inform public health action and prevent additional deaths. The death certification reference guide released in October 2017 by the National Center for Health Statistics informs medical examiners and coroners about completing death certificates for disaster-related deaths (*6*). Public health practitioners can refer to this document to understand current disaster-related death certification processes and how they can collaborate with medical examiners and coroners to obtain the specific mortality data needed to shape disaster-related public health communication strategies. Many public health agencies use traditional surveillance systems and social media surveillance to collect near real-time morbidity and mortality data. The more accurate and thorough the information, the more specifically communicators can target vulnerable groups with appropriate prevention messages.

Because circumstance of death typically provides more detailed information than cause of death, using circumstance of death for disaster-related mortality data analysis is more likely to guide public health action. A single cause of death might be associated with multiple circumstances of death. For example, a cause of death such as “blunt force trauma” could be associated with a motor vehicle crash or being struck by a falling object. The specific circumstances can inform different prevention messages. However, abstracting circumstance of death from the death certificate is more challenging than ascertaining the cause of death. Whereas “Cause of Death” is a labeled field in the death certificate, circumstance of death is determined through assessment of information in other free-text fields within the death certificate, such as “How Injury Occurred.”

Literature on U.S. hurricane-related mortality from recent decades has categorized circumstances of death (*8*,*9*). However, because each disaster is unique, the level of detail available in the circumstance-of-death categories varies across these reports. Not all disasters require a detailed analysis of all death circumstances; however, subcategorization of the most prevalent circumstances of death might reveal additional information that can be used to inform public health messages and interventions. For example, the circumstance of exacerbation of an existing medical condition might include subcategories such as stress-induced, disruption of emergency medical services, and power outage.

Public health messaging about hurricane safety and prevention of hurricane-related injuries should be communicated effectively in the hurricane preparation and response phases (*10*). With a sound understanding that death circumstances can be subcategorized and that categories might overlap, public health practitioners can perform supplemental analyses that will inform more specific and effective public health messaging and interventions to reduce disaster-related injury and illness. By looking at overlapping circumstances of death, analysis of Hurricane Irma mortality data revealed two unique subcategories of heat-related and oxygen-dependent deaths in which power outage contributed to exacerbation of an existing medical condition. Deaths associated jointly with power outages and existing medical condition exacerbation can be minimized by prioritizing power restoration to locations with vulnerable populations, including elderly persons and those with chronic diseases who are especially prone to heat-related illness. In addition, public health messages emphasizing generator safety and widespread use of carbon monoxide detectors can help reduce power outage–related carbon monoxide poisoning.

The findings in this report are subject to at least one limitation. The data might not include all deaths related to Hurricane Irma. As during any disaster, delayed reports of indirectly related deaths might not be recorded because of the imposed end dates of disaster-related mortality surveillance. In addition, death certifiers might change and refine circumstances of death as new information becomes available after registration of the death certificate; these death records are not included. Understanding the need for subcategorization of disaster-related circumstances of death can help public health practitioners develop more effective public health interventions to prevent deaths in future disasters.

**Summary**

**What is already known about this topic?**

Collecting and analyzing mortality data is important for understanding the main circumstances of deaths related to a disaster such as Hurricane Irma.

**What is added by this report?**

Among deaths attributed to Hurricane Irma, the most common circumstance-of-death categories were exacerbation of existing medical conditions and power outage. Further analysis revealed two unique subcategories of heat-related and oxygen-dependent deaths in which power outage contributed to exacerbation of an existing medical condition.

**What are the implications for public health practice?**

Understanding the need for subcategorization of disaster-related circumstances of death can help public health practitioners develop more effective public health interventions to prevent deaths in future disasters.
