# *Erratum:* Vol. 67, No. 30

**DOI:** 10.15585/mmwr.mm6812a5

**Published:** 2019-03-29

In the report “Deaths Related to Hurricane Irma — Florida, Georgia, and North Carolina, September 4–October 10, 2017,” on page 829, the sixth sentence of the fifth paragraph should have read “Fourteen (10.9%) of the heat-related deaths occurred among geriatric patients with existing chronic diseases who resided in **a nursing home** in Florida that was without power for several days during a period of hot weather after the hurricane’s landfall.” In addition, on page 831, the second footnote of the figure should have read “^†^Fourteen of the 17 heat-related deaths occurred in residents of **a nursing home** in Florida that was without power for several days.”

